# Vocational identity in decision-making for terminating/continuation of pregnancy following non-invasive prenatal testing and hypothetical diagnosis among Japanese university students

**DOI:** 10.1371/journal.pone.0309537

**Published:** 2024-08-30

**Authors:** Shodai Sunagozaka, Atsuro Tsutsumi

**Affiliations:** 1 Graduate School of Human and Socio-Environmental Studies, Kanazawa University, Kanazawa, Japan; 2 Institute of Transdisciplinary Sciences for Innovation, Kanazawa University, Kanazawa, Japan; 3 University of the Philippines Open University, Los Banos, the Republic of the Philippines; National Research Centre, EGYPT

## Abstract

The use of prenatal testing in Japan is expected to increase. However, there are ethical concerns regarding pregnancy termination upon the detection of fetal chromosomal abnormalities, such as Down syndrome. Furthermore, factors associated with decision-making following a positive result of Down syndrome after prenatal screening remain unclear. Therefore, we aimed to evaluate the association between decision-making among university students following a hypothetical positive result of Down syndrome in prenatal screening and their perception of their future career development defined as vocational identity. This cross-sectional study included 256 individuals (109 men, 143 women, and four who preferred not to answer). Self-anonymous semi-structured questionnaires were distributed to collect information regarding socio-demographic characteristics, vocational identity, and decision-making following a positive prenatal screening result of Down syndrome. Vocational identity was assessed using the Vocational Identity Measure. Women students were more likely to intend to continue the pregnancy (76.9%, p < 0.05); however, students without siblings (68.2%, p < 0.01) and men and women students with higher scores for vocational identities who were raised in an academically oriented family were more likely to intend to terminate the pregnancy (p < 0.05). Therefore, gender and vocational identity were associated with decision-making following a positive result of Down syndrome. Further qualitative and quantitative studies on factors associated with decision-making following a positive result of Down syndrome are necessary to eliminate the burden and social barrier, including gender division of labor and the effect of vocational career for people wishing to parent a child with Down syndrome.

## Introduction

Down syndrome (DS) is the most prevalent chromosomal anomaly causing intellectual disabilities among live-born infants. Its prevalence is associated with higher maternal age [1,2]. Fetal chromosome anomalies, including DS, can be diagnosed using various prenatal tests, which differ based on fetal invasiveness, sensitivity, specificity, and appropriate gestational age for testing [3]. Regarding the prevalence of DS, approximately 0.1%, 0.3%, and 0.2% of live births had DS worldwide and in China and Japan, respectively [4–7]. Non-invasive prenatal testing (NIPT) is a prenatal testing method that has been used in Japanese clinical settings since 2013 and in commercial settings in most countries [8,9]. As of April 2014, NIPT was available in over 60 countries, including developed and developing countries in all six continents [10]. The uptake rate of NIPT in developed countries is high (42.3% in the Netherlands) [11]; however, people living in rural areas in developing countries have little access to prenatal care, and the use of NIPT depends on the level of education, income, and other socio-demographic background [12–14].

Prenatal testing, such as NIPT, is only offered at the request of pregnant women, and the fee is not covered by universal health coverage in Japan [8]. These social and political situations reduce the use of prenatal testing in Japan [15].

However, the increase in the mean age of mothers with their first child is expected to increase the rate of prenatal testing, including NIPT, in Japan [16]. Despite the anticipated increase in the number of prenatal tests conducted in Japan and advances in reproductive medical technology, there is a need to consider potential political, social, cultural, and ethical ramifications [17]. For example, there is an ethical concern that expansion of NIPT and pregnancy termination following positive DS results will result in decreased support for persons with DS and other disabilities and increase the stigma against disabilities [18,19]. However, pregnancy termination following a positive DS result can also be alternatively considered from the perspective of the socio-economic, institutional, environmental, and psychological barriers to raising a child with disabilities.

Moreover, only a few studies have investigated factors contributing to women’s decision-making following a prenatal DS diagnosis, and studies on men are rare [20].

Studies have investigated hypothetical and actual circumstances involving a fetal DS diagnosis or other chromosomal abnormalities among pregnant women, their partners, and individuals without direct pregnancy-related experience [20]. These studies have shown that abundant financial resources [21], older maternal age [22], and lower academic background [23] are associated with the decision-making regarding pregnancy continuation. Moreover, perceived difficulties in caring for a child with DS are among the strongest predictors of using prenatal tests and selective termination [22]. However, a previous study focusing on older maternal age has limitations regarding statistical analysis [22]. Collectively, decision-making following a prenatal DS diagnosis and perceptions regarding parenting a child with DS, educational background, and financial resources are associated with pregnancy termination.

Parenting a child with DS may affect the parents’ careers, which is a factor to consider. A study on the experience of parents who decided to continue the pregnancy after a prenatal DS diagnosis reported that they were more concerned about their child’s future rather than about their career when making their decision [24]. However, different individuals, including the younger generation who lack pregnancy-related experience, may consider their careers more during their decision-making. Therefore, it is crucial to eliminate the burden for the young generation who wish to pursue a vocational career and intend to continue the pregnancy based on evidence.

To our knowledge, a quantitative study on decision-making following prenatal diagnosis and perception of the career has not been conducted. Therefore, we aimed to explore the relationship between decision-making following a prenatal DS diagnosis among Japanese undergraduate students and their perceptions regarding careers, as well as demographic variables, such as the existence of siblings in a hypothetical situation.

## Material and methods

### Participants

This study was conducted between January 2022 and February 2022 at a university in Japan. We recruited regular undergraduate students since they were of reproductive age and faced career decisions after graduation. The inclusion criteria were students registered as regular undergraduate students, and the exclusion criteria were those registered as non-regular students. To identify and select participants, we asked a few faculty members in charge of lectures based on snowball sampling to distribute questionnaires to the students during lectures. Five of the six consulted faculty members agreed to distribute the questionnaires in their classes. To collect the data from diverse backgrounds regarding their majors, we approached classes conducted in different schools, including ones for introductory education, natural science and technology, and social science.

During the lectures, the research author provided a brief oral presentation of the study objectives using a paper-based explanation form in a face-to-face class. For online classes, the explanation form was sent via email. Participants were asked to complete the online questionnaire form during or after the class following a specific deadline. The accuracy of answers in online classes was secured since the anonymity of answers in sensitive topics, such as decision-making following prenatal testing, is more secure in online data collection. We could not confirm the total number of people approached in this study since some students were absent and refused to receive an email regarding online classes. Information that could identify individual participants during or after data collection was not stored.

### Measures

This study was performed using a cross-sectional design. An anonymous self-administered survey was conducted using an online questionnaire (Google form), both online and face-to-face classes, to collect information regarding the participants’ socio-demographic characteristics, vocational identity, decision-making regarding prenatal testing, and experience with abortion. There was no missing value since the online questionnaire form only accepts the data once all the questionnaire items are answered appropriately. The questionnaire comprised 28 items and was completed in 5–10 min.

We collected the following socio-demographic characteristics: gender, school year, major, high-school graduation region, experience of taking a secondary school entrance examination, existence of siblings, and having close persons with disabilities. We did not confirm the severity of the disabilities of close persons. In Japan, it is not mandatory to take secondary school entrance examinations; moreover, public secondary school entry does not require an examination. However, highly academic schools require applicants to pass entry examinations. Therefore, the parents of children who take secondary school entrance examinations are considered to strongly desire their children to have educational achievement [25]. Consequently, participants who had undertaken the secondary entrance examination were considered to be raised in an “academically oriented family.”

Vocational identity was assessed using the vocational identity measure (VIM). The reliability of VIM was confirmed (α = 0.97). In addition its validity was confirmed since the total score of VIM was positively associated with other related scale (My Vocational Situation) (r = 0.72, p < 0.01) [26,27]. Vocational identity is defined as knowledge regarding stable patterns of career interests, goals, and abilities; moreover, it emerges from integrating life experiences [27]. Therefore, each item in the scale was developed based on this definition. The VIM comprises 20 items evaluated using a 5-point Likert scale (1 = strongly disagree, 5 = strongly agree), with two reverse-worded items. The total VIM score was calculated based on the average score of all items, and no cut-off point was set. VIM is used since it measures stable vocational identity rather than one point and is cited in the public health study as a theoretical model [28]. The original VIM version is written in English; however, most participants’ mother tongue was Japanese. Therefore, we obtained written permission to conduct back-translation based on the normative academic method from the corresponding author of the VIM by email [26]. Researchers in the fields of linguistics, career development, public health, and psychology conducted the translation.

Furthermore, we assessed the decisions made following a hypothetical prenatal DS diagnosis through NIPT within > 10 gestational weeks using the following options: “continue pregnancy,” “terminate pregnancy,” and “not sure.” In the analysis, “not sure” and “continue pregnancy” were categorized as “no intention to terminate pregnancy,” whereas “terminate pregnancy” was categorized as “intention to terminate pregnancy.” The experience of terminating a pregnancy by the participants themselves or their partners was also determined using the following options: “yes,” “no,” and “prefer not to answer.”

We focused on decision-making based on the participants’ current knowledge regarding disabilities and personal values; therefore, we did not provide or assess information regarding DS, social welfare for parents of children with DS, genetic counseling, and deciding on whether to undergo prenatal testing.

### Data analysis

Categorical variables among socio-demographic characteristics were analyzed using the χ2-test between participants with intention to terminate a pregnancy and those with no intention to terminate a pregnancy. The scores for all items were reported and analyzed using unpaired *t*-tests between two groups to examine the relationship between VIM score and decision-making following prenatal testing in detail. Multiple regression analysis was also conducted. The outcome variable was decision-making with the intention to terminate a pregnancy or not, and explanatory variables were set as gender, the existence of close persons with disabilities, the existence of siblings, the total VIM score, and being raised in an academically oriented family.

All statistical analyses were performed using Statistical Package for the Social Sciences software for Mac, version 27 (Chicago, State of Illinois). Statistical significance was set at *p* < 0.05. We did not obtain permission from the participants for the public use of the collected data; therefore, the data cannot be shared in the public data repository.

### Ethical considerations

This study involved sensitive matters, including the personal experience of abortion and the value of the lives of persons with disabilities, which may cause negative emotional reactions from the participants. Therefore, potential participants received information regarding the negative aspects of participating in this study, the study’s purpose, methods, lack of relevance of participation or responses to the class grade, voluntary participation, and the right to withdraw at any time. Written informed consent was obtained in the first item of the questionnaire. Additionally, to secure the confidentiality of the responses, the participants completed the questionnaire on their smartphones since they are much smaller than computers or paper-based questionnaires, which prevented other participants from seeing the responses. Participants were also provided with the contact address of the principal investigator in case they felt emotionally stressed. Therefore, they could contact the principal investigator for support and access to appropriate medical resources, such as student counseling services. Ethical approval was obtained from the Institute of Human and Social Sciences Ethics Committee of Kanazawa University (approval number: 2021–58).

## Results

Among the 257 individuals who completed the questionnaire, we included 256 individuals (109 men, 143 women, and four who preferred not to answer) who met the inclusion criteria in the analysis. In total, 78 individuals (30.4%) intended to terminate the pregnancy, and 159 (69%) did not intend to terminate the pregnancy. This study’s total number of participants was not confirmed since some students were absent and refused to receive an email regarding online classes.

Table 1 shows that gender (p < 0.05) and the existence of siblings (p < 0.01) were significantly associated with the decision to terminate the pregnancy. Men participants (40.9%) and participants without siblings (68.2%) were more likely to intend to terminate the pregnancy. However, the location of the high school where participants graduated from, years in school, having a major, having close persons with disabilities, and experience with pregnancy termination were not significantly associated with the decision-making.

**Table 1:** Demographic variables and the association of the decision-making to terminate the pregnancy following a positive DS result.

	No Intention to Terminate Pregnancy		Intention to Terminate Pregnancy			
	Frequency	%	Frequency	%	χ^a^	
Gender					11.1	*
Men	65	59.1	45	40.9		
Women	110	76.9	33	23.1		
Prefer not to answer	4	100.0	0	0.0		
High-school region of graduation					6.1	
Hokuriku area	97	54.2	29	62.8		
Other than Hokuriku area	82	45.8	49	37.2		
Undergraduate academic year					4.3	
First	145	81.5	59	75.6		
Second	16	9.0	6	7.7		
Third	12	6.7	9	11.5		
Fourth	5	2.8	3	3.8		
Fifth	0	0.0	0	0.0		
Sixth	0	0.0	1	1.3		
Academic Major					4.7	
Humanity and social science	111	62.0	42	53.8		
Natural science and engineering	38	21.2	26	33.3		
Medicine, pharmacy, and nursing science	30	16.8	10	12.8		
Raised in an academically oriented family					0.46	
Yes	29	83.1	14	17.9		
No	145	16.9	64	82.1		
Existence of persons with disabilities who is close to the participants					1.1	
Yes	46	27.0	26	33.3		
No	128	73.0	52	66.7		
Existence of sibling(s)					12.2	**
Yes	162	93.3	17	21.8		
No	12	6.7	61	68.2		
Experience with pregnancy termination					0.8	
Yes	1	1.3	1	1.3		
No	171	96.1	75	96.2		
Prefer not to answer	6	2.6	2	2.6		

* *p* < 0.05

** *p* < 0.01.

^a^ = Chi-squared test.

Table 2 shows the distribution between decision-making following a positive DS result and the VIM score of participants raised in an academically oriented family and their comparison based on gender. The table regarding total participants shows a significant association between decision-making following a positive DS result and one VIM item (16: I have a pretty good sense of what type of work I would like to be doing when I leave school.) (*p* < 0.05). A lower VIM score was associated with the decision to continue the pregnancy. Regarding analysis based on gender, statistical significance was observed for VIM items in both men and women (15: I cannot make a decision about what I want to do for a living, 8: I have no problem deciding what I want to do for a living.) (*p* < 0.01, *p* <0.05, respectively). Cohen’s d for the total VIM score in all, men and women participants were 0.41, 0.68, and 0.01, respectively.

**Table 2:** Distribution of the decision to terminate the pregnancy following a positive DS result and VIM of participants who were raised in academically oriented families.

		Whole						Men						Women					
		No Intention to Terminate Pregnancy		Intention to Terminate Pregnancy		*t* ^*a*^		No Intention to Terminate Pregnancy		Intention to Terminate Pregnancy		*t* ^*a*^		No Intention to Terminate Pregnancy		Intention to Terminate Pregnancy		*t* ^*a*^	
		Mean	SD	Mean	SD			Mean	SD	Mean	SD			Mean	SD	Mean	SD		
1	It is clear to me what I want to do for a living and that I have the right abilities to do well in it.	3.13	0.82	3.00	1.15	-0.32		3.00	0.67	2.89	1.36	-0.23		3.11	0.81	3.20	1.79	0.18	
2	I know what occupational path I want to pursue when I get out of school.	3.07	0.94	2.93	1.44	-0.33		3.10	1.10	2.78	1.30	-0.59		3.11	0.88	3.20	1.79	0.18	
3	I have a clear sense of my occupational interests.	3.53	0.97	3.29	1.33	-0.70		3.70	0.95	3.11	1.17	-1.21		3.42	1.02	3.60	1.67	0.31	
4	I could easily describe my ideal job to a recruiter.	3.20	1.10	3.14	1.23	-0.16		3.50	1.18	3.11	1.17	-0.76		3.11	1.05	3.20	1.64	0.16	
5	I know what type of work I would like to do for the rest of my life.	3.57	1.07	3.14	1.35	-1.12		3.70	1.06	3.00	1.12	-1.40		3.40	1.07	3.40	1.82	-0.03	
6	I have a strong sense of who I am related to the world of work.	2.93	0.91	3.14	1.23	0.64		3.20	0.99	2.89	1.05	-0.69		2.79	0.92	3.60	1.52	0.73	
7	My interests match my vocational goals.	3.40	0.97	2.86	1.41	-1.49		3.30	0.95	2.56	1.13	-1.56		3.58	0.84	3.40	1.82	-1.44	
8	I have no problem deciding what I want to do for a living.	3.40	1.13	3.71	1.07	0.87		3.80	1.03	3.33	1.12	-0.95		3.26	1.15	4.40	0.55	2.28	*
9	I have a firm sense of what type of work I would like to do for a living.	3.53	0.94	3.36	1.22	-0.53		3.80	0.92	3.11	1.17	-1.44		3.32	0.89	3.80	1.30	-0.09	
10	I am having a difficult time choosing what type of work I would like to do. ^b^	3.23	1.10	3.43	1.22	0.53		2.70	0.82	3.11	1.05	0.95		3.42	1.12	4.00	1.41	0.98	
11	I know which type of occupation I would enjoy doing in the future.	2.87	0.97	2.86	1.23	-0.28		3.00	1.16	2.56	1.24	-0.81		2.74	0.87	3.40	1.14	1.42	
12	I have made a firm decision regarding what I want to do for a living.	3.30	0.95	3.00	1.36	-0.85		3.40	1.08	2.56	1.24	-1.59		3.16	0.83	3.80	1.30	1.36	
13	I know what kind of work suits me best.	2.80	0.96	2.50	1.02	-0.95		2.70	1.16	2.33	1.00	-0.73		2.79	0.86	2.80	1.10	0.02	
14	I can readily envision what kind of work I want to be doing when I graduate.	3.33	0.92	2.86	1.23	-1.43		3.40	0.97	2.89	1.27	-0.99		3.37	0.90	2.80	1.30	-1.15	
15	I cannot make a decision about what I want to do for a living.^b^	2.53	1.04	3.07	1.21	1.52		1.90	0.74	3.22	1.09	3.12	**	2.79	1.03	2.80	1.48	0.02	
16	I have a pretty good sense of what type of work I would like to be doing when I leave school.	3.97	0.49	3.14	1.29	-2.31	*	3.80	0.63	3.00	1.23	-1.82		4.00	0.33	3.40	1.52	-0.88	
17	I feel that the vocation of my choice will be the best possible fit for me.	3.17	0.87	1.08	1.08	-1.72		3.00	0.82	2.78	1.09	-0.51		3.21	0.92	2.40	1.14	-1.68	
18	I feel like I am on a definite vocational path for the future.	3.37	0.96	3.07	1.00	-0.94		3.60	0.97	2.89	0.93	-1.63		3.32	0.95	3.40	1.14	0.17	
19	I have certain vocational goals that I would like to pursue when I get out of school.	3.33	0.96	2.79	1.19	-1.63		3.40	0.97	3.00	1.23	-0.80		3.26	0.99	2.40	1.14	-1.68	
20	It is clear to me what I want to do for a living after I graduate.	3.56	0.90	2.79	1.31	-2.01		3.40	0.74	2.67	1.23	-1.46		3.47	0.91	3.00	1.58	-0.89	
	Total Score of VIM	3.28	0.64	2.99	0.89	-1.25		3.41	0.84	2.86	0.90	-1.48		3.21	0.61	3.22	0.92	0.03	

* *p* <0.05

** p < 0.01.

VIM = Vocational Identity Measure; SD, standard deviation.

a = t-test.

b = Inverted Items, score is reversed.

Table 3 indicates the result of logistic regression analysis. In addition to variables with statistical significance observed in Table 1, other related ones including the total score of VIM were chosen as explanatory variables in logistic regression analysis. Being a woman and having siblings had significantly higher odds ratios in decision-making with the intention to continue the pregnancy (2.41 and 3.82, respectively). In addition, not having close persons with disabilities, a higher VIM score, and being raised in academically oriented families led to decision-making with the intention to terminate a pregnancy. Naglkerle R^2^ was 0.129.

**Table 3:** Logistic regression analysis on decision-making with the intention to terminate the pregnancy following a prenatal DS diagnosis (n = 256).

Outcome: decision-making with the intention to terminate pregnancy or not^a^				
Independent variables		OR	95% CI	
Gender ^b^		2.41	0.489–2.206	**
Whether one has close persons with disabilities		0.73	0.392–1.341	
Whether one has siblings		3.82	1.660–8.775	**
Total VIM score		1.30	0.906–1.864	
Whether one was raised in an academically oriented family		1.04	0.63–1.30	

** *p* < 0.01.

Naglkerle R^2^ = 0.129.

^a^ Intention to terminate pregnancy coded 0, no intention to terminate pregnancy coded 1.

^b^ Men coded 0, Women coded 1, prefer not to answer coded 2.

OR = odds ratio; CI = confidence interval.

## Discussion

Following a prenatal DS diagnosis using NIPT, we examined the association of decision-making with vocational identity and socio-demographic characteristics. We found that men students and students without siblings were likely to terminate the pregnancy. Moreover, men and women students with higher VIM scores, who were raised in academically oriented families, were likely to terminate the pregnancy,.

In our study, the rate of the decision to continue the pregnancy was relatively low compared with previous findings in hypothetical [22,29–31,37] and real-world situations [32–36]. For instance, a real-world study conducted in Turkey reported that employed mothers, those with high income, and families with educational backgrounds significantly terminated pregnancies when DS was identified in a fetus [35]. Another study that examined the termination rate based on ethnic group and the seven common fetal aneuploidies in the United States indicated that the termination rate among Hispanic (70%) and Filipino (80%) pregnant women is lower than that among Caucasian (90%), African American (92%), and Asian women (87%) [36]. These studies were hospital-based and reflect actual situations regarding decision-making following prenatal testing. However, the examination of factors associated with decision-making is limited, especially in terms of expectations related to parents’ careers, such as vocational identity.

Furthermore, our findings regarding gender differences in the decision to terminate the pregnancy differ from previous findings. A study on pregnant women and their men partners using NIPT in Japan showed that more pregnant women intended to terminate the pregnancy if the NIPT result was positive when compared with the men partners [37]. This difference could be attributed to the differences in decision-making between hypothetical and actual situations following a prenatal DS diagnosis.

Therefore, this difference suggests that the decision to continue the pregnancy following a positive DS result in a hypothetical situation may change in a real-world situation when facing the concrete burden of continuing the pregnancy and gender divisions of labor.

For example, in Japan, the time for which women engage in unpaid work, including parenting, is five times more than that among men [38]. Further, another study showed that the primary caregiver of a child with DS is the mother [39]. Efforts are being made to support children with DS, including inclusive education in Japan [40]; however, several emotional stresses, such as multidimensional courtesy stigma against parents of children with DS, have been reported in Japan [41,42].

Regarding our findings of significant differences based on the existence of sibling(s), a previous study conducted in the UK investigated the attitude of women who have siblings with DS toward pregnancy termination after prenatal testing [22]. Similar to result with previous study, this study found that people with a siblinghad varying perceptions toward pregnancy termination after prenatal testing. Therefore, further studies are warranted on the influence of siblings on this decision.

We found that a higher score for one VIM item among men and women students who were raised in an academically oriented family was significantly associated with the decision to terminate the pregnancy. It seems that the item associated with the ability to decide what they do as a vocational career by themselves was significantly associated with decision-making. Furthermore, people with a clear image of a future career, as indicated by a higher VIM score, consider having a child with DS as negatively affecting their career. Additionally, having a child with DS, who often has learning disabilities, may lead to being exposed to an environment different from that in which the academically oriented family was raised.

This study has some limitations. First, there was a choice to answer “not sure” regarding decision-making following a positive DS result, which was categorized as “no intention to terminate a pregnancy”. In real-world settings, “not sure” is not an applicable decision and may result in a decision to terminate a pregnancy rather than "no intention to terminate,” as assumed in the present study. Furthermore, this study was conducted at one university using snowball sampling and an online questionnaire approach with a cross-sectional study design, and there is a moderate selection bias regarding academic majors. Therefore, comparative studies among universities inside or outside Japan with more question items, including those on socio-economic status and cultural background, with quantitative and qualitative studies, may be essential to ensure further generalizability. In addition, power analysis should be conducted to identify the appropriate number of participants in advance.

Despite the above limitations, this is, to our knowledge, the first study to investigate decision-making following a positive DS result among university students in Japan. Therefore, this study will contribute to further studies and practices in eliminating factors associated with gender and vocational careers that could lead to pregnancy termination after a positive DS result.

## Conclusion

Gender, the existence of siblings, and vocational identity were associated with decision-making following a positive DS result. However, further qualitative and quantitative studies on factors associated with decision-making following a positive DS result are necessary to confirm the validity of this study and eliminate the burden and social barrier for people wishing to parent a child with DS and pursue their vocational career.
